# Numerical and Experimental Analysis of Strength Loss of 1.2709 Maraging Steel Produced by Selective Laser Melting (SLM) under Thermo-Mechanical Fatigue Conditions

**DOI:** 10.3390/ma16247682

**Published:** 2023-12-17

**Authors:** Jarosław Piekło, Aldona Garbacz-Klempka, Dawid Myszka, Krzysztof Figurski

**Affiliations:** 1 Faculty of Foundry Engineering, AGH University of Krakow, Mickiewicza 30, 30-059 Krakow, Poland; jarekp60@agh.edu.pl; 2 Faculty of Mechanical and Industrial Engineering, Institute of Manufacturing Technologies, Warsaw University of Technology, 85 Narbutta Street, 02-524 Warsaw, Poland; dawid.myszka@pw.edu.pl; 3 Saga Poland, Ltd., Cieszyńska 23 G, 43-170 Łaziska Górne, Poland; kfigurski@saga-mb.eu

**Keywords:** thermal fatigue, SLM method, numerical analysis, metallography analysis, die casting

## Abstract

The result of the development of additive manufacturing (AM) methods is the increasing use of the selective laser melting (SLM) method as a technique for producing tooling for injection moulds and die casting pressure moulds from maraging steel powders. The mould components are subjected to varying thermo-mechanical loads during these operations. This paper presents a numerical model that is used to predict the fatigue life of a material that is loaded with a time-varying temperature field according to the classic and modified Coffin test. Using a computational model, the temperature changes in the resistance-heated specimen and the stress and strain fields that are caused by this phenomenon were determined. Using three different multiaxial fatigue criteria, the fatigue life of SLM steel was determined. Numerical calculations were verified using experimental thermal fatigue tests on 1.2709 SLM steel that was aged at 490 °C as well as via metallographic tests. The numerical model was used to predict the durability of the same steel aged at 540 °C. The effect of specimen clamping conditions on the fatigue life of SLM steel was determined numerically. The value of the decrease in strength of SLM steel as a result of the increasing number of cycles of temperature changes was determined experimentally; a great influence of ageing temperature on fatigue life was found. Changes in the structure of steel occurring during cyclic changes in temperature are presented.

## 1. Introduction

Three-dimensional printing methods offer new possibilities in the processes of constructing machine parts and tooling. Thanks to the process of making parts from layers that are welded together, this greatly expands the space of design solutions. These methods make it possible to produce parts that cannot be made by cavity machining or casting methods. One of the possibilities of using incremental methods using maraging steel powders as a material is to make toolings for injection moulds and die casting moulds; the selective laser melting (SLM) method is most commonly used for this purpose [1]. The reason for applying this unconventional method to the production of mould parts is the possibility of making a cooling system that is optimal in terms of heat transport in a mould [2,3,4,5,6]. The cooling system in a mould part that was made using the SLM method can consist of channel sections with almost any trajectory and cross-sectional shape (as opposed to a traditional system that consists only of rectilinear sections). Some physical and mechanical properties of maraging steel that is produced using the SLM method differ from those of this type of steel when made using traditional methods. The reason for the differences that occur is the specifics of the SLM process. The issues that are related to the selection of SLM process parameters, microstructures, heat treatments, and strength testing of 1.2709-type maraging steel produced via the SLM method have been described in many publications. The authors of numerous publications have agreed that a too-high laser beam layer-scanning speed and a too-high powder-layer thickness result in a decrease in material density and an associated decrease in hardness [7], strength, and ductility [8,9]. Solidification of the molten powder occurs through a cellular–dendritic growth mechanism, while the very high cooling rate during the SLM process results in no martensite laths being observed in the microstructure [10]. On the metallographic specimens, characteristic lines that represent the outlines of the molten metal lake are visible. Immediately after the SLM process, maraging steel has a too-low hardness for those parts that are made from it and are intended to be used as pressure mould parts. Hence, heat treatment involving ageing within a temperature range of 490 °C to 540 °C is usually applied. As a result of this ageing, intermetallic phases are released that strengthen the alloy, causing increases in the hardness and strength while decreasing the ductility [11,12,13,14]. The kinetics of the process of separating the intermetallic phases in SLM steel during ageing as well as their chemical composition, size, and morphology are not fundamentally different from those occurring during the heat treatment of the same grade of steel that is produced conventionally [15]. In the case of the use of parts made by the SLM method as casting elements of pressure moulds, properties of the printed material such as its strength at elevated temperatures and its resistance to thermal fatigue caused by cyclic temperature changes become particularly important. Due to the complexity of the phenomena occurring during the exploitation of devices that are subjected to a varying temperature field, it is difficult to develop a universal method for testing the material’s resistance to thermal fatigue. It is usually assumed that thermal fatigue is the process of the formation and development of cracks in the material and changes in its properties that are caused by the action of repeated temperature changes. The typical feature of thermal fatigue is an increase in material volume that is caused by the accumulation of micro-crack volumes (which are induced by temperature changes). In structural components, there is usually an overlap of purely thermal influences and mechanical loads resulting from the limitation of thermal deformation by various types of bonds. Thermo-mechanical fatigue then occurs, which is also characteristic of pressure mould parts. Therefore, an important issue before conducting experimental tests and defining the numerical model is to determine and analyse the effects of thermal and mechanical factors on the durability of the material during its operation (in this case, during the operation of a pressure mould). Issues that are related to analyses of the stress and strain field that are caused by cyclic temperature changes during the operation of pressure moulds have been the subject of many studies and publications [16,17,18,19,20]. The results of the research and the calculations of the authors of this work were used to establish the conditions for the experiment and define the calculation models. Taking the operating conditions of those parts of the mould that are in contact with the liquid metal into account, the experimental study was carried out using a modified Coffin test. During the test, one end of the specimen was rigidly clamped, while the other clamping fixture had controlled stiffness so that the specimen could deform in the direction of its axis. As mentioned earlier, the subject of the test was 1.2709 maraging steel that was obtained via the SLM method. According to the Raju [21], the fatigue strength of SLM steel is lower than that of the same steel grade that is obtained via traditional methods. The authors’ research with a high-cycle fatigue test (HCF) with a cycle asymmetry factor of R = −1 showed that a significant increase in the fatigue strength of steel after printing can only be achieved by combining a properly selected heat treatment with surface machining and that the direction of the specimen’s axis relative to the work platform during the printing affects the value of the fatigue limit. Samples whose axes were perpendicular to the work platform had a significantly lower fatigue limit when compared to those whose axes were parallel to the surface of the base plate. In his study, Mower [22] showed that when the axes of printed samples are parallel to the plane of the base plate, the fatigue strength of 316L SLM steel was only 5 to 15% lower than that of forged steel. The tests on 18Ni300 SLM steel at HCF and R = 0 that were conducted by Branco [23] showed a slight weakening effect of the material during the test and identified surface and internal defects in the form of unmelted metal as fatigue crack-initiation sites. In his study, Wang [24] indicated that internal defects in the material resulting from the SLM process caused bifurcation of the main crack and a deviation of the propagation of the main crack from the direction of the maximum load. According to Fatemia’s research presented in [25], unavoidable internal defects in the material that were created during the technological process and the directionality of the structure had a significant impact on the course of multiaxial fatigue. In this case, the notch effect that was caused by internal defects in the material added up to a multiaxial stress state and had a negative effect on the fatigue strength. Far fewer publications present the results of thermal and thermo-mechanical fatigue tests on SLM steels. In his study, Wang [26] compared the thermal fatigue resistance of forged H13 steel that was produced using the SLM method and was also used to make pressure moulds. The specimens were heated and cooled in the area of the notched crack—the end of which was the crack initiator. A greater thermal fatigue resistance of SLM steel was found; according to the authors, the reason for this was the much smaller grain size (which counteracted the development of fatigue cracks in the vicinity of the crack). Also, the scanning strategy affected the length of the propagating crack in the material after being loaded with a variable temperature field. In the case of the same scanning direction in different layers, the structure of the metal was orderly and promoted the development of long cracks, while changing the scanning direction created a random grain orientation and counteracted the propagation of long cracks [27]. Thermal fatigue resistance tests have been carried out using various methods—the results of which can only be compared with each other within the scope of one method. Hence, testing has been carried out under industrial conditions in some cases. An example of such a method was the installation of a test plate directly in a pressure casting mould [28]. The test plate was placed in contact with injected metal. After a certain number of metal injections, it was removed from the mould for analysis on its surface of crack initiation and propagation.

The purpose of the study presented in this article was to investigate the effect of temperature cycling on the mechanical strength and fatigue life of maraging steel used for SLM die casting parts. Taking into account the objective difficulties in carrying out industrial tests, the material was tested on a laboratory test bench. The range of temperature changes during the laboratory tests corresponded to the temperature changes in the surface of the pressure mould cavity. On specimens subjected to a varying number of thermal fatigue cycles, the strength drop was determined each time in the tensile test. The authors found an initial significant decrease in strength, which they attribute to an increase in the proportion of retained austenite as determined by the EBSD method and to changes in the structure of the steel visible on metallographic images between 1 and 1000 cycles. Due to the time-consuming nature of thermal fatigue testing (one cycle lasts about 60 s), a numerical model of thermal fatigue on test specimens was developed to determine the fatigue life of SLM steels under near-actual mould operating conditions. The results obtained contribute to the elucidation of material destruction processes occurring in the surface layers of the pressure mould cavity and to the prediction of strength and fatigue life degradation of SLM steel tested, subjected to different heat treatment variants.

The article is organised as follows. Section 2 collects the main information on the properties of the material studied, the equipment used, and the experimental procedure. Section 3 and Section 4 present the analytical and numerical model used to predict the fatigue life of SLM steel. Section 5 provides the results of numerical calculations based on the presented computational models. Section 6 presents the results of experimental investigations to determine the strength degradation of steel induced by temperature cycling and changes in the microstructure. The final section presents an analysis of the results of the calculations and experimental tests.

## 2. Materials and Methods

The specimens for the thermal fatigue and strength testing were made using the SLM method from 1.2709 steel powder with the trade name MS1 (recommended by EOS). The samples were fabricated via SLM using an M2 Concept Laser Cusing device at Industrial Development Agency S.A. in Radom, Poland. The system was characterised by 1070 nm Ytterbium (Yb) fibre laser of maximum laser power (2 × 400 W) and scan speed (7000 mm/s). The preheating of the substrate was not applied. The samples were printed under a protective atmosphere of nitrogen. During the printing, the axes of the samples were located perpendicularly to the plane of the working platform. The scanning strategy was to divide each powder layer into square islands, which were to be scanned by the laser beam in a random manner. The layer thickness was 25 µm, the laser power was 180 W, the hatch space was 90 μm, and the scanning speed was 1100 mm/s.

The chemical composition of the alloys was verified using a SPECTROMAXx emission spectrometer with z iCAL 2.0. Chemical composition of the alloy of SLM-printed samples is given in Table 1.

**Figure 1:**
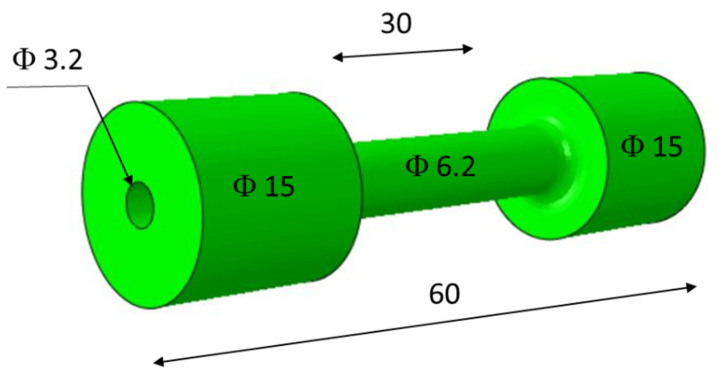
Shape and dimensions (mm) of thermal fatigue test specimen.

The average relative density of the samples measured using the Archimedes method was 99.93% ± 0.04. Cylindrical specimens with a length of 60 mm were used for thermal fatigue testing. The measuring part of the specimen was 30 mm long and 6.2 mm in diameter. Along the axis of the specimen, there was a hole with a diameter of 3.2 mm. The shape and dimensions of the thermal fatigue test specimen are shown in Figure 1.

**Figure 2:**
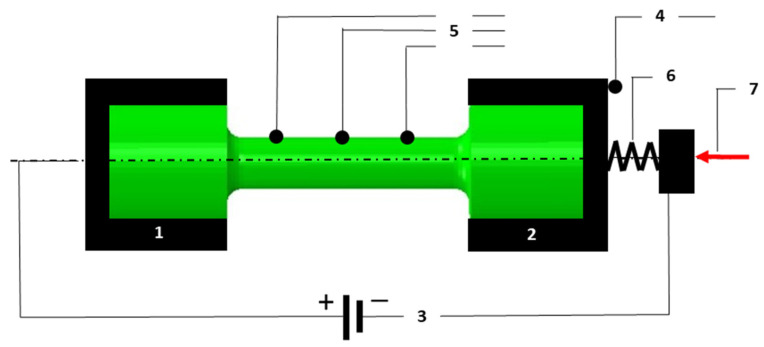
Schematic of thermal fatigue test stand: 1—stationary clamp; 2—moving clamp; 3—current supply; 4—displacement sensor; 5—temperature measurement system; 6—element of constant stiffness; and 7—actuator regulating the clamping force acting on component “6”.

The specimens were heat-treated by heating at 100 °C/h and withstanding at 490 °C (HT490) and 540 °C (HT540) for 6 h, followed by cooling at 100 °C/h. In order to strengthen the alloy due to the formation of intermetallic phases, ageing was carried out in a microprocessor-controlled “Mini Tube KJ 1200” furnace with a diameter of 40 mm and a length of 200 mm. Thermal fatigue tests were carried out based on a modified Coffin method on a dedicated test stand [29]. The classic Coffin test involves the cyclic heating and cooling of specimens that are fixed in rigid grips. In our modified Coffin test, one of the grips in which the specimen was mounted had the ability to move along the specimen axis thanks to the controlled stiffness of the fixture. The test consisted of cyclic heating of the specimens to 600 °C and then cooling to 200 °C. The time of one cycle was 60 s. Heating the specimen to 600 °C took 10 s. Resistive heating of the samples was carried out by the flow of a current of 10 A. During the test, the temperature changes in the surface of the specimen and the displacement of the movable grip that held the specimen were recorded. A simplified schematic of the measurement system for the thermal fatigue testing and measuring the displacement and temperature of the specimen is shown in Figure 2.

The number of heating and cooling cycles of the specimen was counted and recorded using a cycle counter. Thermal fatigue tests were conducted up to a predetermined number of heating and cooling cycles without cracking the specimens. After recording the required number of cycles, each specimen was subjected to a tensile test on an MTS 810 tensile testing device. During the conducted test, changes in the force F and elongation of specimen u were recorded. It was assumed that the maximum force Fmax that was recorded during the test was a measure of the thermal fatigue-induced destruction of the specimen material. A reference value of the material strength was also determined on specimens that were not subjected to thermal fatigue. The number of cycles of temperature change followed by the decohesion of the material due to only thermal fatigue (F = 0) was also determined. Metallographic studies of the fracture surfaces and cross-sections of the samples were carried out using a TESCAN MIRA (Brno, Czechia) scanning electron microscope with an FEG electron source, equipped with an EBDS detector by Symetry S2 (produced by Oxford Instruments, Abingdon, UK). To reveal the microstructure, a mixture of picryl acid and alcohol ethyl was used to etch the samples.

## 3. Coupled Stress/Thermal-Electrical Model of Phenomenon

A coupled thermal-electrical numerical model was developed to determine the temperature changes that would occur in the sample during the thermal fatigue testing. The heating of the specimen to a certain temperature was caused by a current flow of 10 A and the Joule–Lenz effect that this flow caused. The thermal-electrical model and the other numerical models were prepared in Abaqus CAE v. 2019 software environment [30]. The material definition routines, calculation algorithms, and finite element database that were available in this program were used. Table 2 and Table 3 summarise the electrical, thermal, and strength properties of 1.2709 SLM steel aged at 490 °C and 540 °C that were used in the numerical model.

The geometric model of the sample was discretised using 20-node DC3D20E quadratic coupled thermal-electrical brick elements. The number of elements in the sample model for each type of numerical analysis was the same at 62,935 elements. Changes in the temperature field in the sample were determined via coupled thermal-electric analysis. The heat-transfer coefficients between the surface of the sample model and the environment were corrected based on experimental temperature measurements on the sample surface; the end result was a correspondence between the numerical solution and the measurement data. Based on the calculated values of the temperature in the sample during one heating and cooling cycle, the displacement, strain, and stress field occurring in the sample model were determined. Changes in the strain–stress field in the sample were determined using general static analysis. The calculations were carried out for variants of the classic Coffin test (with the rigid mounting of both ends of the specimens) and the modified Coffin test (when one of the grips in which the specimen was mounted had the possibility to move due to the controlled stiffness of the clamping). In this case, the accuracy of the numerical calculations was verified by comparing the calculated and experimentally measured displacement of the end of the specimen. Twenty-node quadratic brick elements with reduced integration C3D20R were used to discretise the stress model.

## 4. Thermal Fatigue Model

The fatigue lives of the specimens that were made via the SLM method from 1.2709 steel powder were also determined via a numerical method using Durability Analysis Software for Finite Element Models fe-safe v.2019 [31]. The calculations were carried out for the classic and modified Coffin tests. The fatigue calculations were carried out using the following hypotheses: “Normal Strain” (N-S); “Brown-Miller” (B–M); and “Maximum Shear Strain” (Max-Sh-St). The fatigue cycle was determined on the basis of the courses of the changes in the stresses and strains of the specimens that were determined using the Abaqus program. The coefficients appearing in the fatigue life equations were determined using the Seeger approximation [31] on the basis of the data that were obtained from the tensile tests within a temperature range of 200 to 600 °C. The following relationships were present in the Seeger approximation: fatigue strength coefficient σ_f_′ = 1.5 UTS; fatigue ductility coefficient ε_f_′ = 0.59a and a = 1.375–125 (UTS/E); and strain hardening coefficient K′ = 1.65 UTS. In the Seeger approximation method, coefficients b and c had constant values: fatigue strength exponent b = −0.087; and fatigue strain exponent c = −0.57. The N-S criterion assumes that a fatigue crack develops perpendicularly to the plane, on which the amplitude of principal strain Δε_1_ is the greatest. For a uniaxial stress state, the maximum magnitude of the principal strain vector has the same direction as the maximum axial strain. Cycles of normal strain Δε_1_ are extracted from the strain tensor; the algorithm uses the strain–life curve that is defined by the following equation:

(1) $$\frac{{∆\varepsilon }_{1}}{2}=\frac{\sigma_{f}^{\prime }}{E}{\left({2N}_{f}\right)}^{b}+\varepsilon_{f}^{\prime }{\left({2N}_{f}\right)}^{c}.$$

The B–M criterion is based on the concept of the critical plane and assumes that fatigue failure occurs in the plane where the greatest amplitude of shear strain Δγ_max_ occurs and that the fatigue life is a function of both the shear strain γ_max_ and strain ε_n_ that are perpendicular to the critical plane. The fatigue life calculation algorithm is defined by the following equation:

(2) $$\frac{{∆\gamma }_{max}}{2}+\frac{{∆\varepsilon }_{n}}{2}=1.65\frac{\sigma_{f}^{\prime }-\sigma_{m,n}}{E}{\left({2N}_{f}\right)}^{b}+1.75\varepsilon_{f}^{\prime }{\left({2N}_{f}\right)}^{c},$$

σ_m,n_ is the mean normal stress on the plane.

The third Max-Sh-St criterion that is considered assumes that cracks initiate on the plane that has the maximum amplitude of shear deformation. In this case, the fatigue life is calculated using the following formula:

(3) $$\frac{{∆\gamma }_{max}}{2}=1.3\frac{\sigma_{f}^{\prime }}{E}{\left({2N}_{f}\right)}^{b}+1.5\varepsilon_{f}^{\prime }{\left({2N}_{f}\right)}^{c}.$$

The values of the coefficients occurring in Equations (1)–(3) for the SLM steel (HT490 and HT540) are summarised in Table 4 and Table 5.

## 5. Numerical Results

### 5.1. Temperature Field

**Figure 3:**
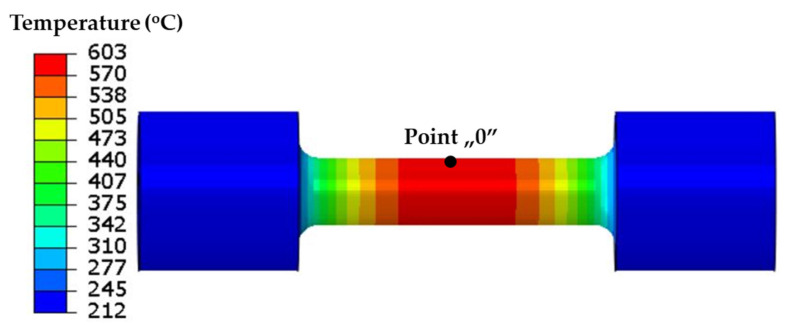
Temperature on surface of sample model after 10 s of resistance heating.

The current temperature model was used to calculate the value of the temperature field in the sample. Figure 3 shows the temperature field on the surface of the sample at the 10 s mark of the cycle when the temperature in the middle of the sample reached a maximum value of 600 °C.

**Figure 4:**
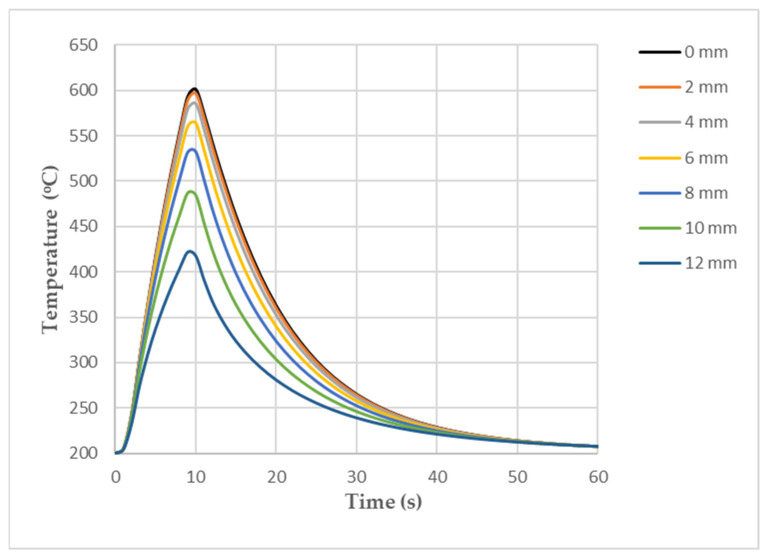
Temperature changes in surface of sample model at point “0” and at points 2, 4, 6, 8, 10, and 12 mm away from it.

Figure 3 shows the symmetry of the temperature field and the change in the temperature values occurring along the axis of the sample, which was caused by the cooling of the grips in which the sample was mounted. The temperature changes in the surface during the heating and cooling phases of the sample at the “0” point (Figure 3) and at points symmetrically located at distances of 2, 4, 6, 8, 10, and 12 mm from the “0” point are shown in Figure 4.

The temperature difference between the centre of the sample and the end of the sample in the vicinity of the grips was about 200 °C (as can be seen in the temperature change curves that are shown in Figure 4).

### 5.2. Classic Coffin Model

**Figure 5:**
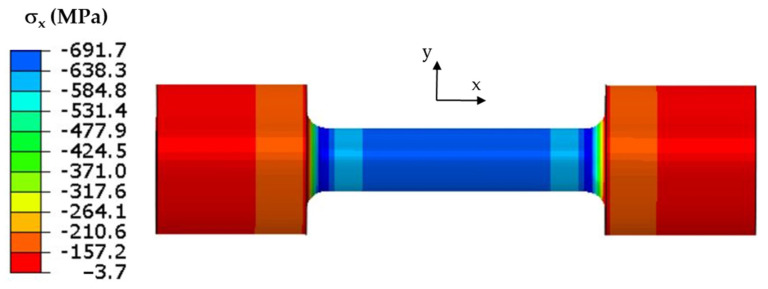
Axial stress σ_x_ at end of heating specimen with both ends stiffly fixed.

The classic Coffin test assumes no displacement of either end of each specimen. Cyclic temperature changes in a specimen induce stresses. The stress and strain values in the specimen model were determined from the temperature changes in the model that were described above. The field of maximum axial stress values σ_x_ at the end of the resistance-heating phase when the temperature at the “0” point (Figure 3) was 600 °C is shown in Figure 5.

**Figure 6:**
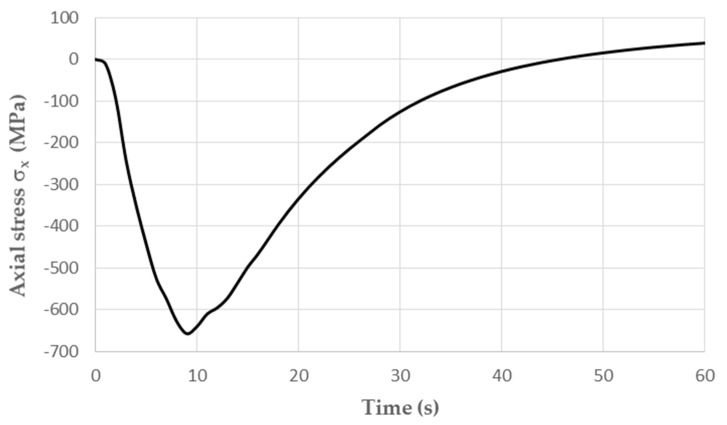
Changes in axial stress σ_x_ in specimen at point “0” with rigid attachment of both ends occurring during one fatigue cycle.

When the temperature reached 600 °C in the middle part of the specimen, compressive stresses σ_x_ of about −670 MPa dominated. The remaining circumferential and radial stress components did not exceed 20 MPa. As the temperature decreased, the compressive stresses also decreased; by the end of the cycle, they changed to positive figures, and the centre of the specimen was tensile (Figure 6). The maximum tensile stress in the fatigue cycle was σ_x_ = 40 MPa.

The description of the simultaneous action of mechanical strains and loads resulting from the change in temperature is possible only because both actions induced corresponding states of strain. The basic equation describing this relationship is the Duhamel–Neumann equation, which can be written in a simplified form for the case under consideration [32]:

(4) $$\varepsilon_{tot}=\varepsilon_{\sigma }+\alpha ∆T,$$

where ε_σ_ strain that is induced by the state of stress, α—the coefficient of the linear expansion, ΔT—the temperature change, and ε_tot_—the total strain. The course of cyclic deformation resulting from the superposition of the mechanical and thermal loading is conveniently presented in the following form:

(5) $$\varepsilon_{tot}=\varepsilon_{M}+\varepsilon_{T},$$

where thermal strain *ε_Τ_* = *α*Δ*T*, and mechanical strain *ε_Μ_* = *ε_σ_*. For a rigidly constrained specimen, Equation (25) takes the following form (assuming a uniform temperature distribution along its length):

(6) $$\varepsilon_{T}-\varepsilon_{M}=0.$$

**Figure 7:**
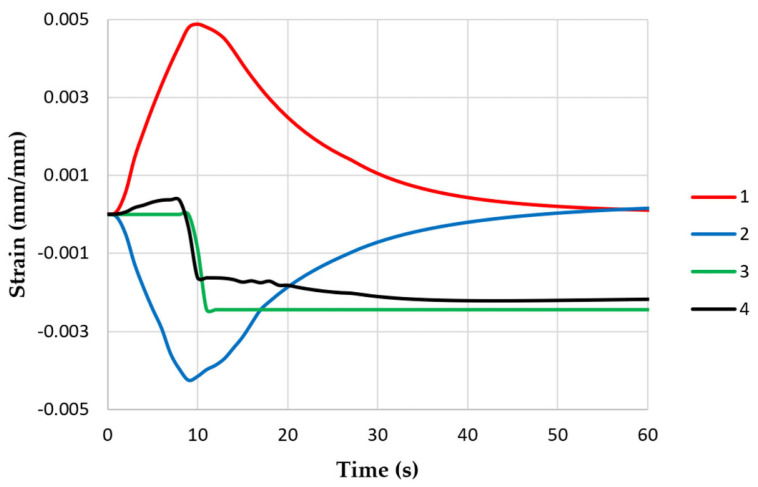
Changes in strain values in middle parts of specimen models (with both ends of each rigidly fixed): 1—thermal strain ε_Τ_; 2—elastic strain ε_e_; 3—plastic strain ε_pl_; and 4—total strain ε_tot_.

Omitting some of the deformations that are associated with the creep phenomenon, one can write the following:

(7) $$\varepsilon_{M}=\varepsilon_{pl}+\varepsilon_{e},$$

where *ε_pl_* is the plastic strain, and *ε_e_* is the elastic strain. The strain changes in the middle parts of the specimen models are shown in Figure 7. Thermal deformation caused plastic and elastic deformation. At the end of the thermal fatigue cycle, neither the stress nor the strain returned to their initial values. This fact was taken into account when calculating the fatigue lives of the specimens.

### 5.3. Modified Coffin Model

A modification of the classic Coffin model features the introduction of controlled clamping stiffness. During the thermal fatigue test, one end of the specimen (together with the clamping fixture) has the possibility of limited displacement in the direction of the specimen axis. The value of the maximum displacement of the specimen that is caused by temperature changes depends on the value of the clamping stiffness (c ≠ ∞ or c ≠ 0). With the introduction of the above assumptions on the specimen clamping, Equation (6) will take the following form:

(8) $$\varepsilon_{T}-\varepsilon_{M}\neq 0$$

**Figure 8:**
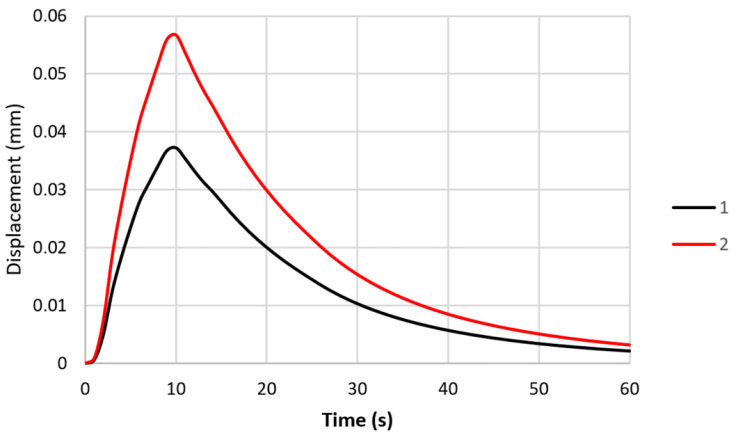
Displacement of moving ends of specimens during one heating and cooling cycle: 1—maximum displacement of 0.037 mm; and 2—maximum displacement of 0.057 mm.

Using the numerical model, the states of the stress and strain in the specimen were determined for two different specimen clamping stiffnesses. An indirect measure of the stiffnesses in the analysed models was the value of the displacements of the ends of the specimens, which could be compared with the displacements that were recorded during the experimental testing. The changes in the displacements of the ends of the specimens for the two numerical models that were analysed are shown in Figure 8. During the experimental tests, the maximum displacements of the ends of the specimens were 0.037 and 0.057 mm.

**Figure 9:**
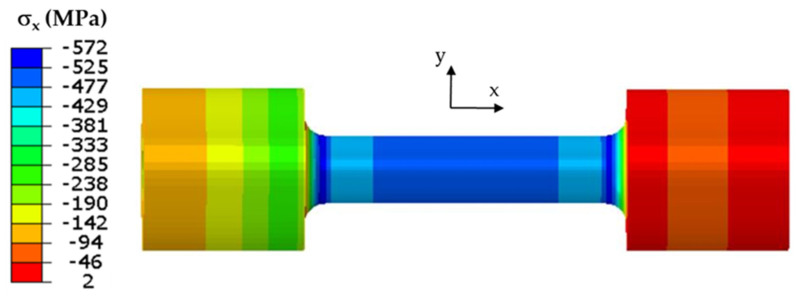
Axial stress σ_x_ at end of heating of specimen in model with controlled clamping stiffness; maximum displacement of end of specimen was 0.037 mm.

The stress field on the surface of the specimen model for which the maximum end displacement along the *x*-axis was 0.037 mm is shown in Figure 9.

**Figure 10:**
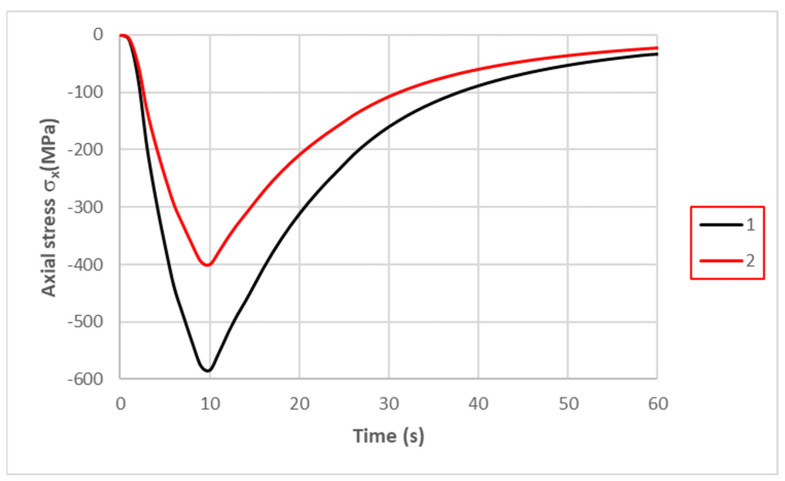
Changes in axial stress σ_x_ during one cycle in specimen models with controlled clamping stiffnesses occurring during one fatigue cycle: 1—maximum displacement of 0.037 mm; and 2—maximum displacement of 0.057 mm.

The greater stiffness of the specimen clamping affects the magnitude of the generated stresses. Figure 10 shows plots of the changes in axial stress σ_x_ at the centres of the specimens when the maximum displacements of the ends were 0.037 and 0.057 mm. The greater stiffness of the specimen clamping resulted in the smaller displacement of the end of the specimen and an increase in the value of the axial stress for the same heat loading.

**Figure 11:**
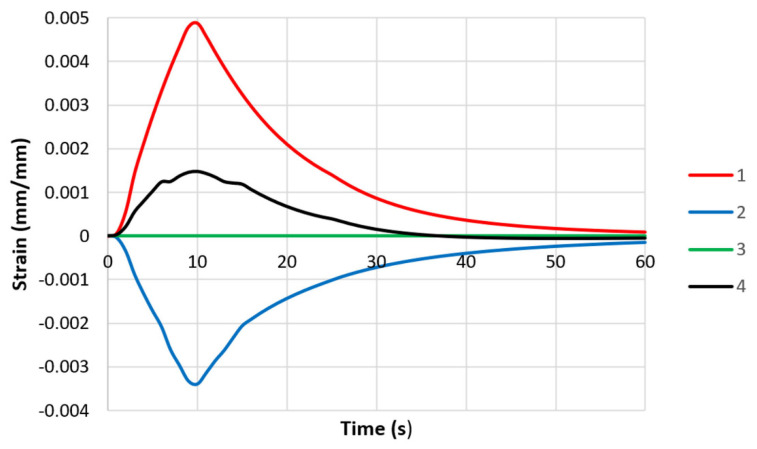
Changes in strain values in middle parts of specimen models with controlled clamping stiffnesses: 1—thermal strain ε_Τ_; 2—elastic strain ε_e_; 3—plastic strain ε_pl_; and 4—total strain ε_tot_ (maximum displacement—0.037 mm).

**Figure 12:**
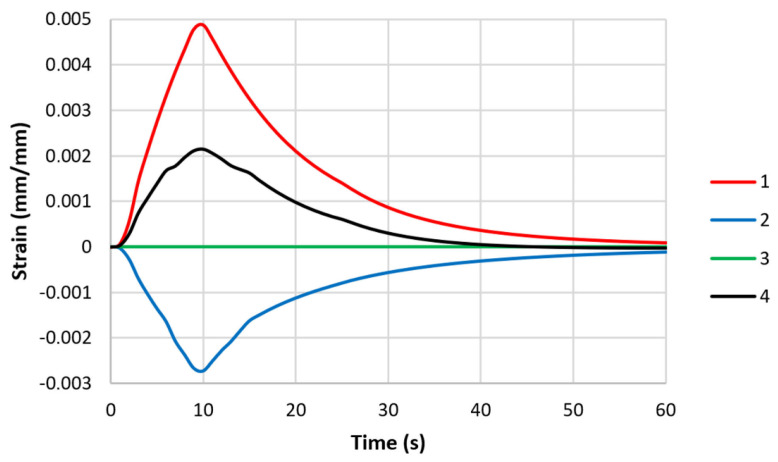
Changes in strain values in middle parts of specimen models with controlled clamping stiffnesses: 1—thermal strain ε_Τ_; 2—elastic strain ε_e_; 3—plastic strain ε_pl_; and 4—total strain ε_tot_ (maximum displacement—0.057 mm).

Unlike the model with the rigid attachment of both ends of the specimen, there was no plastic deformation in either of the models with controlled stiffness (Figure 11 and Figure 12). The thermal deformation was the same in both models with varying stiffnesses and caused only elastic deformation.

### 5.4. Fatigue Life Prediction for 1.2709 SLM Steel

**Figure 13:**
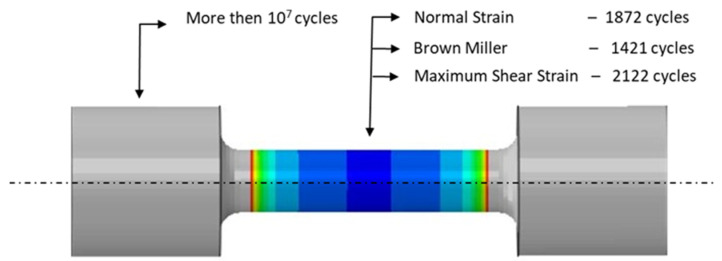
Model of constrained double-sided HT490 post-treatment specimen for thermal fatigue testing; crack location marked with arrows; and numbers of cycles for crack initiation calculated according to N-S, B–M, and Max-Sh-St criteria. In zones of equal colour, the number of cycles for crack initiation is the same.

Calculations to determine the number of fatigue cycles after exceeding the point where crack initiation occurs were carried out using the fe-safe program. The fatigue cycle was defined by the states of the stress and strain in the specimen occurring at three time points of a cycle: at the beginning, at the moment of the maximum strain and stress, and at the end of the cycle. In the computational model, the surface roughness was assumed to be Ra = 20 μm. Figure 13 shows a model of the specimen with a colour map plotted on its surface showing the numbers of cycles that initiated cracks. The calculations were carried out for a material with the properties of SLM steel aged at 490 °C. The specimen model was constrained at two ends, and the number of cycles was calculated based on three fatigue criteria based on the critical plane concept. The figure shows the numbers of crack-initiating cycles that were calculated according to the N-S, B–M, and Max-Sh-St criteria.

The same calculation methodology was used to predict the fatigue lives of specimens that were clamped with controlled stiffnesses and featured maximum specimen end displacements of u = 0.037 and 0.057 mm. The results of the calculations are summarised in Table 6.

Similar calculations were performed for material that was aged at 540 °C (Table 7).

The simulations showed that the method for clamping the specimen that was adopted in the model had a very strong effect on the number of cycles that initiated fatigue cracking: the greater the stiffnesses of the grips that held the specimen, the greater the values of the stresses that were generated, and the lower the numbers of cycles that preceded crack initiation. Note the large difference between the numbers of cycles that initiated the cracking of the rigidly fixed specimens (u = 0) and those that had the possibility of slight displacements along their axes. In the case of the HT490 heat treatment, the difference amounted to one order of magnitude (e.g., 912 and 9816 cycles—Table 7); this was even two orders of magnitude for HT540 (e.g., 1872 and 167,787 cycles—Table 6). The calculations also showed the quite large effect of the ageing temperature of the steel (defined in the numerical models by the choice of material coefficient values) on the fatigue life of 1.2709 SLM steel. The heat treatment of HT490 that was carried out at a lower temperature resulted in the greater fatigue resistance of the SLM steel than when ageing HT540 at the higher temperature.

## 6. Test Results

### 6.1. Mechanical Testing

**Figure 14:**
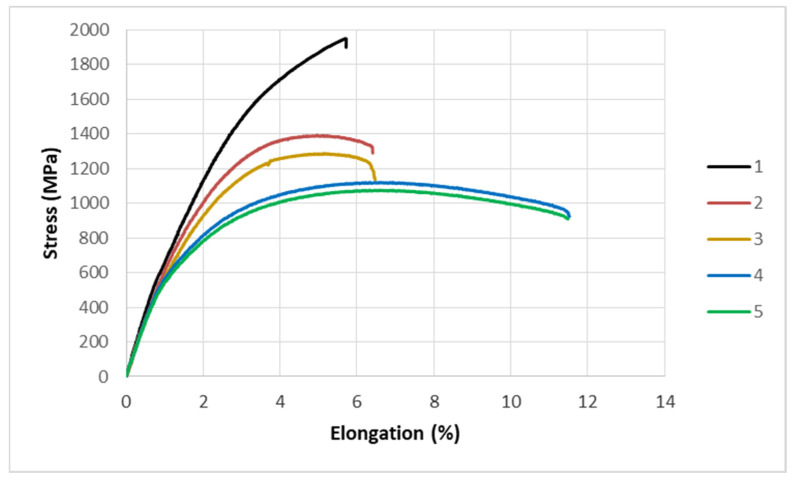
Typical tensile curves of specimens that were previously subjected to different numbers of thermal fatigue cycles: 1—reference sample; 2—100 cycles; 3—3000 cycles; 4—8000 cycles; and 5—16,000 cycles.

The mechanical testing consisted of subjecting samples that were made via SLM from 1.2709 steel powder and aged at 490 °C to an alternating temperature field that was induced by a current flow. The middle part of the specimen was heated to 600 °C and then cooled to 200 °C. The tests were carried out with the controlled stiffness of one of the grips, allowing for a maximum displacement of 0.037 mm at the end of the specimen. When a predetermined number of fatigue cycles was reached, the test was stopped, and the specimen was subjected to a tensile test. During the static tensile test, the elongation and F-force were recorded. The exceptions were the reference specimens (which were only subjected to tensile testing—number of cycles = 0) and those that failed directly during the thermal fatigue cycles (destructive force F = 0). Figure 14 shows the typical plots that were obtained during the tensile test of HT490 heat-treated SLM steel specimens that were previously subjected to thermal fatigue within a range of 100 to 16,000 cycles along with the reference specimen.

Table 8 shows the results from the tensile tests of the 1.2709 SLM HT490 steel that was subjected to different numbers of thermal fatigue cycles. Due to the time-consuming nature of the tests, the results are the average values of only two tests. The differences in the obtained values of failure force F did not exceed ±0.2 kN in any case. The fracture of all of the tested specimens occurred exactly in the plane of symmetry; that is, where the temperature changes were the greatest during the thermal fatigue.

The tests that were carried out showed that, after 100 fatigue cycles, there was a significant reduction in the strength of the material (from 1949 to 1390 MPa). The subsequent fatigue cycles resulted in smaller decreases in strength (decreasing by 18 MPa between 500 and 5000 cycles). A significant strength decrease of 160 MPa was observed between 5000 and 8000 fatigue cycles. The subsequent fatigue cycles to which the specimen was subjected did not cause major changes in the strength (Figure 14—curves 4 and 5).

### 6.2. Metallographic Studies

**Figure 15:**
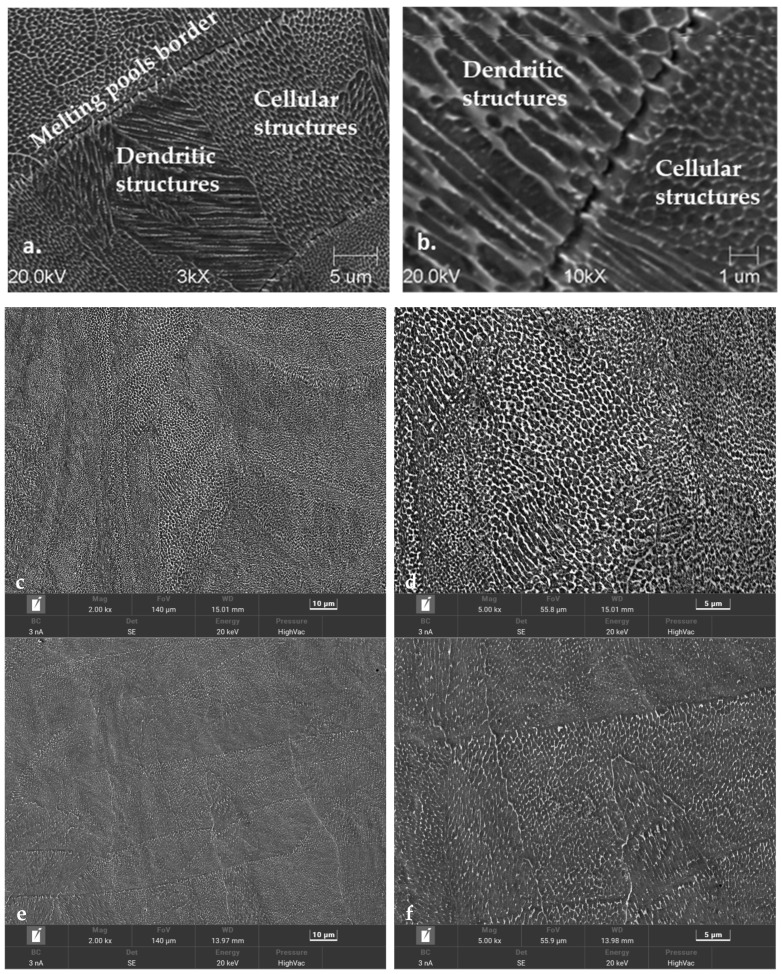

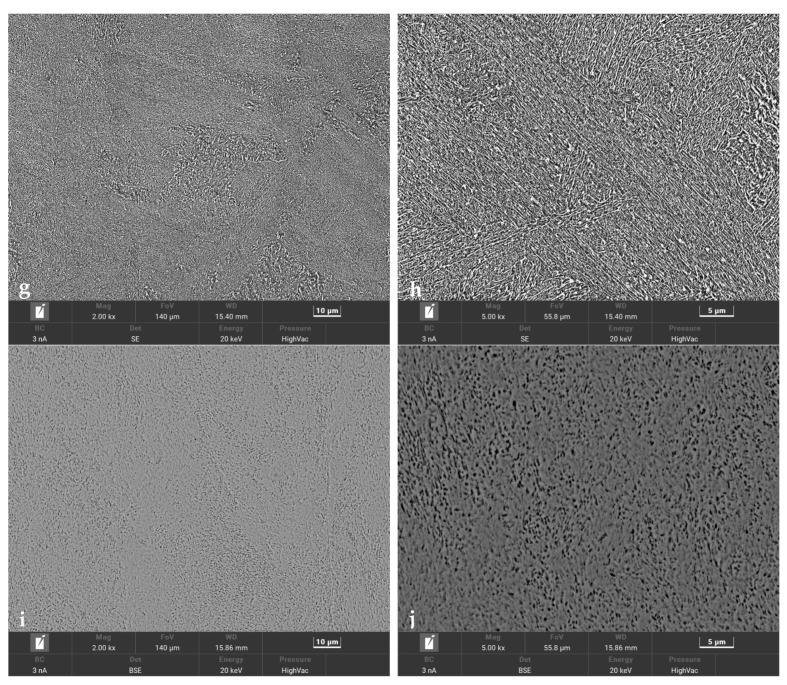
SEM images showing microstructure of SLM-formed maraging steel: (**a**,**b**) as-built condition; (**c**,**d**) HT490; (**e**,**f**) HT490 (+100 heat fatigue cycles); (**g**,**h**) HT490 (+8000 cycles); and (**i**,**j**) HT490 (+16,000 cycles).

The metallographic tests that were carried out on 1.2709 SLM HT490 steel were designed to reveal the changes occurring in the structure as a result of thermal fatigue. After the tensile tests, the specimens were cut along a plane parallel to the specimen axis. As mentioned earlier, the specimen axis was positioned perpendicularly to the surface of the work platform during the printing. The images in Figure 15 show the differences between the structure of the steel after HT490 ageing and temperature cycling and the structure of the as-built condition steel. When observed with the scanning microscope, the as-built condition steel structure showed areas of cellular and dendritic structures as well as boundaries between the grains and the areas of the remelting (Figure 15a,b). The phenomenon of epitaxial cell growth was also visible (Figure 15b). The heat treatment of the steel by ageing HT490 caused the boundaries between the cells and the grains to become less distinguishable, but the scanning images partially revealed their presence in this case (Figure 15c,d). In the scanning images of the aged steels that were subjected to temperature cycling (100 cycles), the grain boundaries and cellular or dendritic structures were practically invisible at different image magnifications (Figure 15e,f). Increasing the number of fatigue cycles (8000 cycles) no longer caused visible changes in the microstructure image (Figure 15g,h). Above 16,000 cycles, the cellular structure of the alloy was no longer visible (Figure 15i,j).

**Figure 16:**
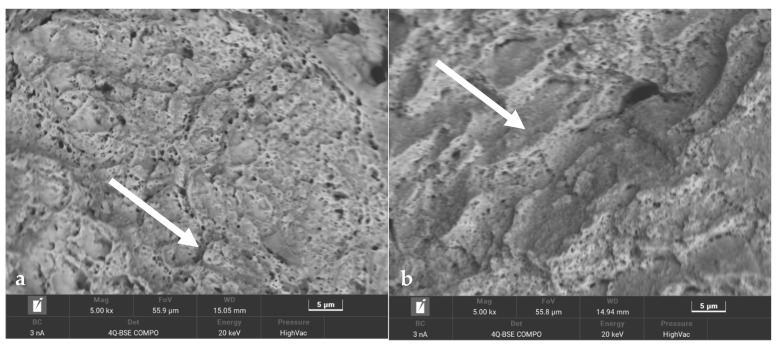
Fragment of fracture surface of maraging steel HT490 after static tensile tests on specimens that had been previously subjected to temperature cycling: (**a**) 100 cycles; areas of plastic zones is marked by the white arrow and (**b**) 8000 cycles; the fracture showed slip zones marked by the white arrow.

The changes in the microstructures of the steels under varying heat loads (shown in Figure 15) resulted in changes in the strengths of the steels. The significant change in the microstructures of the steels that was observed during the first 100 temperature cycles was reflected in a significant decrease in strength (Figure 15c–f). Between 1000 and 8000 temperature cycles, the grain boundaries and cellular structures were still present, but they became progressively less pronounced; this corresponded to a smaller strength reduction in the steels than during the first 100 cycles (Figure 15g,h). From 8000 up to 16,000 cycles, the cell structures and grain boundaries were no longer visible, and the structural images did not differ (Figure 15i,j). Within this range of fatigue cycles, there was very little loss of strength in the SLM steels. The number of thermal fatigue cycles to which the specimens were subjected also influenced the modes of decohesion and the morphologies of the fractures that were obtained after the static tensile testing. Figure 16 shows images of the fracture surface morphology of two specimens from 1.2709 SLM HT490 steel that were previously subjected to 100 and 8000 temperature cycles. The fracture of the steel after 100 cycles had the characteristics of a brittle resolution fracture, with areas of plastic zones in the forms of characteristic dimples; one of these is marked by the white arrow in Figure 16a. Prolonged exposure to varying heat loads increased the ductility of the steel. The fracture (Figure 16b) showed slip zones (marked by the white arrow), and deformation occurred by shearing.

**Figure 17:**
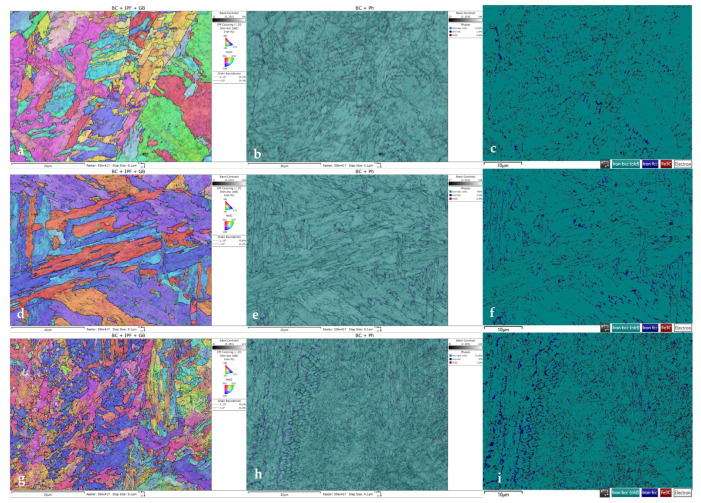
EBSD analysis of vertical cross-sections taken from specimens (**a**–**c**) HT490; (**d**–**f**) HT490 (+100 heat fatigue cycles); and (**g**–**i**) HT490 (+8000 heat fatigue cycles).

The increase in the ductility of 1.2709 SLM HT490 steel is related to austenite reversion, which occurs at temperatures that are above the ageing temperature. Increasing numbers of fatigue cycles results in increases in the proportion of the austenite in the steel structure; this was confirmed via the EBSD tests (Figure 17).

The coarse-grained martensitic structure and random orientation of the grains are visible in the colour inverse pole figure (IPF) in Figure 17a,d,g. Fractions of the retained austenite occurred mainly at the martensite boundaries (Figure 17b,e,h). This result was confirmed by previous studies [33], which showed that the martensite lath boundaries were the location of the austenite reversion and the start of nucleation. It is difficult to make an accurate quantitative assessment of the percentage of retained austenite due to the large grain sizes and the unknown crystallographic texture. Despite these difficulties, Figure 17c,f,i show increases in the proportion of the reverse austenite fraction as the number of thermal fatigue cycles to which the SLM steel was subjected increased. After 100 fatigue cycles, a 93% increase in the fraction of the austenite could be observed; after 8000 cycles, this increased to 166% as compared to its fraction in the SLM steel after heat treatment (HT490). Between 8000 and 16,000 fatigue cycles, no significant increase in the proportion of the reversed austenite could be observed.

## 7. Analysis of Results

Based on laboratory tests and numerical calculations, an analysis of the strength reduction in 1.2709 SLM steel under the influence of temperature cycling was carried out in order to provide information that can be used to predict the fatigue life of pressure mould parts. As presented in the introduction, some parts of moulds are made via the SLM method in order to obtain optimal layouts of cooling channels (which cannot be performed using machining methods). Therefore, the temperature cycling to which the samples were subjected was chosen to approximate the temperature changes in a mould. It was assumed that the contact temperature of a mould surface with a metal alloy during injection is 600 °C [34,35], while the temperature of the mould surface after opening (when the lubricant is sprayed) drops to 200 °C. In addition to the appropriately adjusted temperature changes, a sample wall thickness of 1.5 mm was also assumed to be approximately equal to the thickness of such a mould layer where these temperature changes will occur. The values of the stresses and strains and the number of cycles that cause crack initiation depend on both the range of the temperature changes and the way a specimen is clamped. It is difficult to unambiguously determine the boundary conditions for clamping the specimen in grips that correspond to the boundary conditions that occur in the mould; therefore, a numerical analysis of the stress–strain state was carried out for three variants of specimen end-clamping; the first of these assumed that the specimen was rigidly fixed in both grips, while the second and third conditions allowed one end to move. The condition of both ends being rigidly fixed corresponded to the least favourable conditions when the stresses take their maximum values and the fatigue life is the lowest. The boundary conditions for the clamping of the specimen that assumed that the ends could be displaced by 0.037 and 0.057 mm generated stress values that were comparable to those in mould cavity areas [36]. In this study, the stress–strain state of HT490 steel was analysed using FEM for the three aforementioned specimen-attachment methods. For the models with rigidly fixed ends, maximum axial compressive stresses of −670 MPa occurred when the temperature at the centre of the specimen reached 600 °C. As the temperature decreased, the compressive stresses decreased; by the end of the cycle, this changed to a positive number. In the central part of the specimen, axial tensile stresses of 40 MPa were present (Figure 6). The change in the stress sign was due to the permanent plastic deformation that occurred towards the end of the fatigue cycle (Figure 7). Due to its high stress values and the lowest fatigue life, this was the least favourable case of the mechanical boundary conditions. Stress variations during the thermal fatigue cycle in those models where one end of each specimen was allowed to move were characterised by lower values of maximum axial stress, which were −400 MPa for 0.057 mm and −590 MPa for 0.037 mm end displacements, respectively (Figure 10). The decreases in the clamping stiffness resulted in decreases in the axial compressive stress values. In both cases of the models with controlled clamping stiffnesses, there were no changes to positive in the signs of the stress at the ends of the cycles. The reason for this was the lack of plastic deformation in both models (Figure 11 and Figure 12). The numerically determined changes in the mechanical and thermal strains in the central parts of the specimen models did not satisfy Equation (2) due to the temperature differences along the specimen axis that were caused by the cooling of the ends of the specimens (among other things). Equation (2) assumes a constant temperature value along the entire axis of a heated specimen. The time-consuming nature of the fatigue tests was the reason for reducing the experimental tests to a single case of specimen clamping during the fatigue testing (end displacement of 0.057 mm). The results of these tests were used to calibrate the numerical model. The error in the numerically calculated maximum end displacement of the specimen was found to be no more than 1.1% with respect to the experimentally measured value. Depending on the applied fatigue hypothesis, the numbers of cycles that were determined viav the calculations based on the critical plane concept were 167,787, 82,034, and 174,910 cycles (end displacement of 0.057 mm). The critical plane concept assumes that a fatigue crack is induced by the action of stress or strain in a specific plane of a material. The rationale for this assumption is the experimentally observed occurrence of cracks in metals in certain specific planes. Metallographic studies of fractures have shown that the mode of the decohesion of steel depends on the number of temperature cycles that precede the static tensile test. Above 8000 cycles, the fracture (Figure 16b) showed slip zones (marked with an arrow), and deformation occurred via shearing. In contrast, when each sample was subjected to fewer cycles, the fracture was a mere cleavage with small areas of plastic deformation. The analysis of the results of the numerical calculations showed the strong influence of the way in which the specimen was clamped and, thus, the magnitude of the stresses and strains that were generated within it on the number of cycles that resulted in crack formation. The rigid clamping of the specimens generated the highest values of axial equal stress; plastic deformation was also generated in the material. With this method of specimen clamping, the predicted number of cycles it took to initiate cracking was 2122 for the HT490 heat treatment. When the end of a specimen was allowed to move (controlled clamping stiffness), the predicted fatigue life increased significantly (to 174,910 cycles). The results of the numerical calculations also showed the great effect of the ageing temperature on the fatigue life of 1.2709 SLM steel. A fatigue life analysis was performed on HT490- and HT540-treated steel by varying the material characteristics of the numerical models through the selection of experimentally determined coefficients and material constants (Table 2, Table 3, Table 4 and Table 5). The HT540 treatment caused a significant reduction in the predicted fatigue life of the steel as compared to the HT490 treatment (Table 6 and Table 7); the reason for this was the lower value of the conventional yield strength of the steel that was aged at 540 °C. As mentioned earlier, the investigations and simulations that were carried out in this study were aimed at the possibility of using the results to predict the durability of pressure mould parts that are made from 1.2709 steel powders via the SLM method. The prerequisite for correctly predicting the number of crack-initiating cycles was determining the actual temperature changes in the selected mould parts and the definition of the boundary conditions of the sample fixture; this applied to both the conducting of the experiment and the numerical model. Therefore, any analysis of a similar material strength loss, as was presented in this article, should be preceded by calculations of the temperature changes and stress-state components in a mould. On the basis of the calculated stress value at the selected point in the mould, it is possible to determine the stiffness of the movable support that affixes the specimen; thus, carrying out an experiment or calculation from which the fatigue life of the material can be predicted (in our case, 1.2709 SLM steel). As the numerical models are refined, the parallel use of experimental tests and numerical calculations should increase the latter’s contribution to the life analysis of pressure mould parts.

## 8. Conclusions

The advantages of manufacturing certain parts of pressure mould tooling via the SLM method were reasons for undertaking research to determine the mechanism by which the strength of the printed material decreases under cyclic temperature changes. It is also important to develop a method to predict the number of cycles of temperature change that cause crack initiation. In this study, the most commonly used 1.2709 maraging steel produced via the SLM method for the manufacture of die-cast parts was the subject of our research. Experimental tests that involved subjecting the specimens to temperature cycling were carried out on SLM steel after ageing at 490 °C.

Based on the research, it was shown that

- The strength of 1.2709 SLM steel decreases by about 30% after the first 100 cycles of thermal fatigue; between 500 and 5000 cycles, it remains almost unchanged; between 5000 and 8000 cycles, the strength decreases by 12%; and between 8000 and 16,000 cycles, the decrease in strength is only 4%.
- Metallographic studies have shown a relationship between the structure of steel subjected to temperature cycling and its strength. As the number of cycles increases, the cellular structure of the alloy undergoes progressive degradation. These changes correspond to some extent with the change in strength of the steel. Significant changes in the image of the alloy structure are observed between the 1st and 100th fatigue cycle. In contrast, above 8000 thermal fatigue cycles, the cellular structure of the steel disappears.
- EBSD analysis shows increases in the proportion of the reverse austenite fraction as the number of thermal fatigue cycles, to which the SLM steel was subjected, increased. After 100 fatigue cycles, a 93% increase in the fraction of the austenite could be observed; after 8000 cycles, this increased to 166% as compared to its fraction in the SLM steel after heat treatment (HT490). Above 8000 fatigue cycles, no significant increase in the proportion of the reversed austenite could be observed.
- On the basis of FEM calculations, it was found that the manner in which the specimen is fixed has a strong influence on the fatigue life of the steel. The analysis was carried out in such a range of maximum displacements of the end of the specimen, which generates similar values of stresses that occur at the free surfaces of the pressure mould (u = 0.037) and at the local stress increase caused by the notch effect (u = 0.057) (Table 6 and Table 7).

The numerical model proposed in this paper can be modified according to the type of constraints limiting the thermal deformation of the material and its mechanical properties and used to predict the fatigue life of die-cast moulds.

## Figures and Tables

**Table 1:** Chemical composition wt.% of studied alloy.

Chemical Composition wt.%									
C	Si	Mn	Cr	Ni	Mo	Co	Ti	Al	Fe
0.026	0.11	0.08	0.17	18.4	4.92	9.18	0.82	0.69	Balance

**Table 2:** Mechanical and thermophysical properties describing constitutive model of 1.2709 SLM maraging steel (HT490).

Temperature	UTS (MPa) ^1^	R_p0.2_ (MPa) ^1^	Young’s Modulus (MPa) ^1^	Temperature Range	Linear Expansion Coefficient (1/K) ^2^	Conductivity λ (W/m/K) ^2^	Density ρ (kg/m^3^) ^2^	Specific Heat c (J/kg/K) ^2^	Resistance (Ω × m)
200 °C	1959	1780	190,000	25–200 °C	10.15 × 10^−6^	20	8025	450	6.7 × 10^−7^
300 °C	1886	1703	190,000	25–300 °C	11.50 × 10^−6^				
470 °C	1577	1294	180,000	25–400 °C	11.51 × 10^−6^				
500 °C	1444	1003	162,000	25–600 °C	11.55 × 10^−6^				
600 °C	910	540	120,000						

^1^ Experimental research; ^2^ EOS ToolSteel 1.2709 SLM material data sheet.

**Table 3:** Mechanical properties describing constitutive model of 1.2709 SLM maraging steel (HT540).

Temperature	UTS (MPa) ^1^	R_p0.2_ (MPa) ^1^	Young’s Modulus (MPa) ^1^
200 °C	1742	1623	180,000
300 °C	1620	1510	180,000
470 °C	1156	1016	175,000
500 °C	915	821	156,000
600 °C	535	423	111,500

^1^ Experimental research.

**Table 4:** Coefficients used in numerical calculation of fatigue life for HT490.

Temperature	Fatigue Strength Coefficient σ*_f_*′	Fatigue Ductility Coefficient ε*_f_*′	Strain Hardening Coefficient *K*′ (MPa)
200 °C	2938.5	0.0508	3232.3
300 °C	2829.0	0.0792	3111.9
470 °C	2365.5	0.1651	2602.0
500 °C	2166.0	0.1539	2382.6
600 °C	1365.0	0.2520	1501.5

**Table 5:** Coefficients used in numerical calculation of fatigue life for HT540.

Temperature	Fatigue Strength Coefficient σ*_f_*′	Fatigue Ductility Coefficient ε*_f_*′	Strain Hardening Coefficient *K′* (MPa)
200 °C	2613	0.0975	2874
300 °C	2430	0.1475	2673
470 °C	1734	0.3376	1907
500 °C	1372	0.4363	1510
600 °C	802	0.5921	883

**Table 6:** Predicted fatigue life of SLM HT490 steel expressed in numbers of cycles determined via numerical method using three different hypotheses: N-S, B–M, and Max-Sh-St.

	u = 0 mm	u = 0.037 mm	u = 0.057
N-S	1872	22,098	167,787
B–M	1421	13,476	82,034
Max-Sh-St	2122	24,402	174,910

**Table 7:** Predicted fatigue life of SLM HT540 steel expressed in numbers of cycles determined by numerical method using three different hypotheses: N-S, B–M, and Max-Sh-St.

	u = 0 mm	u = 0.037 mm	u = 0.057
N-S	912	3233	9816
B–M	761	2523	7718
Max-Sh-St	1023	3375	11,545

**Table 8:** Destructive force F_max_, maximum stress σ_max_, and elongation A of 1.2709 SLM HT490 steel subjected to different numbers of thermal fatigue cycles.

Number of Cycles	F (kN)	σ_max_ (MPa)	A (%)
0	43.0	1949	5.59
100	30.7	1391	6.54
500	28.6	1296	8.39
1000	28.6	1296	6.61
2000	28.4	1287	6.47
3000	28.3	1283	6.48
5000	28.2	1278	6.50
8000	24.7	1118	11.50
16,000	23.7	1073	11.60

## Data Availability

The data that support the findings of this study are available from the corresponding authors (J.P., A.G.-K., D.M., K.F.) upon reasonable request.
